# Genetic and diet effects on *Ppar-α *and *Ppar-γ *signaling pathways in the Berlin Fat Mouse Inbred line with genetic predisposition for obesity

**DOI:** 10.1186/1476-511X-9-99

**Published:** 2010-09-10

**Authors:** Asja Wagener, Helge F Goessling, Armin O Schmitt, Susanne Mauel, Achim D Gruber, Richard Reinhardt, Gudrun A Brockmann

**Affiliations:** 1 Humboldt-Universität zu Berlin, Department for Crop and Animal Sciences, Invalidenstraße 42, 10115 Berlin, Germany; 2 Freie Universität Berlin, FB Veterinärmedizin, Institut für Tierpathologie, Robert-von-Ostertag-Straße 15, 14163 Berlin, Germany; 3 Max-Planck-Institut für molekulare Genetik, Ihnestraße 73, 14195 Berlin, Germany

## Abstract

**Background:**

The Berlin Fat Mouse Inbred (BFMI) line is a new mouse model for obesity, which was long-term selected for high fatness. Peroxisome proliferator-activated receptors (PPARs) are involved in the control of energy homeostasis, nutrient metabolism and cell proliferation. Here, we studied the expression patterns of the different *Ppar *genes and the genes in the PPAR pathway in the BFMI line in comparison to physiological changes.

**Results:**

At the age of 10 weeks, the BFMI mice exhibited marked obesity with enlarged adipocytes and high serum triglycerides concentrations in comparison to the often used mouse line C57BL/6 (B6). Between these two lines, gene expression analyses revealed differentially expressed genes belonging to the PPAR pathway, in particular genes of the lipogenesis and the fatty acid transport.

**Conclusion:**

Surprisingly, the *Ppar-α *gene expression was up-regulated in liver and *Ppar-γ *gene expression was down-regulated in the white adipose tissue, indicating the activation of a mechanism that counteracts the rise of obesity.

## Background

The peroxisome proliferator-activated receptors (PPARs) constitute a family of three genes, which are involved in the control of energy homeostasis and cell proliferation. The PPAR family members have distinct patterns of tissue distribution and tissue specific functions. PPAR-α is predominantly present in liver where it has a critical role in the regulation of nutrient metabolism as it stimulates the uptake and oxidation of fatty acids. PPAR-γ is mainly expressed in adipose tissue. It is induced during adipocyte differentiation and is a regulator in the formation of fat cells and lipid accumulation. PPAR-δ is abundantly expressed throughout the body and it has been proposed to be involved in adipogenesis and energy metabolism [for review, see [1-3]]. Activation of PPAR-α by agonists leads to reduced adiposity and lowered triglyceride levels by reduced food intake [4-6], whereas activation of PPAR-γ by agonist stimulates lipid storage and is associated with body weight gain [7,8].

Recently, we have generated the high-fatness selected Berlin Fat Mouse inbred line BFMI as a model for juvenile obesity [9]. BFMI mice harbour natural mutations leading to a five fold increased fat percentage due to hyperphagia at young age and an altered lipid metabolism in comparison to C57BL/6 mice [10,11]. A specific regulation of PPAR genes in the development of obesity in the BFMI mice is very likely.

Therefore, the intention of this study was to investigate whether the Ppars and their responsive genes are involved in the fat accumulation of the BFMI line. Our aims were (i) to identify differences in gene regulation of *Ppars *and their responsive genes between the obese BFMI mouse model and B6 mice as a control and (ii) to analyze responses of PPAR pathway genes to high-fat diet feeding in BFMI mice. Therefore, we analysed transcript amounts of genes belonging to the PPAR pathway in white adipose tissue and liver. Furthermore, we analysed body composition, adipocyte size and serum parameters.

## Results

### Phenotypic differences between lines BFMI and B6

**Figure 1:**
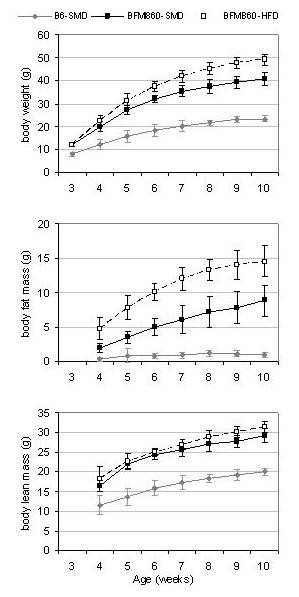
**Comparisons of lines BFMI and B6 on standard maintenance diet (SMD) and of line BFMI on high-fat diet (HFD)**. Body weight, body fat mass and body lean mass from three to 10 weeks of age. Lines differ from 3 weeks of age on and animals between the two diet groups differ from 4 weeks of age in line BFMI (P < 0.05). Each point represents the mean weight with standard deviation.

**Figure 2:**
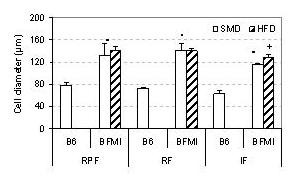
**Adipocyte diameter of reproductive (RPF), retroperitoneal (RF), and inguinal (IF) adipose tissue of B6 and BFMI mice on standard maintenance diet (SMD) and of BFMI mice on high-fat diet (HFD)**. Animals were 10 weeks old.

**Figure 3:**
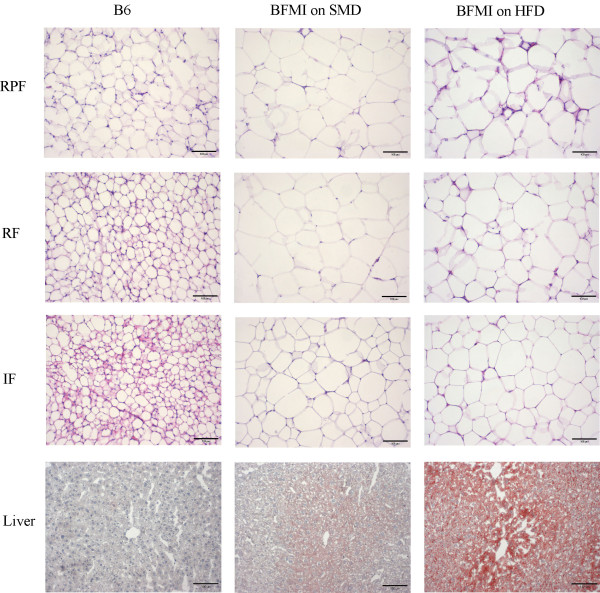
**Hematoxylin and eosin stained section of reproductive (RPF), retroperitoneal (RF), and inguinal (IF) adipose tissue and oil-red staining of liver sections of B6 and BFMI mice on standard maintenance diet (SMD) and of BFMI mice on high-fat diet (HFD) at 10 weeks**.

To compare the body composition of the two lines, male mice of BFMI and B6 were fed a standard maintenance diet (SMD) until week 10. On SMD, animals of the BFMI line were significantly heavier and had more body fat and lean mass than B6 mice over the entire period (Figure 1). At 10 weeks, mice of the BFMI line were 1.8 times as heavy as B6 mice (41.4 ± 2.9 g and 23.5 ± 1.5 g, respectively). The body weight gain was due to increased fat mass. BFMI mice had almost 10 times as much body fat mass but only 1.5 times as much lean mass as B6 animals. In BFMI mice, fat was accumulated in all three investigated adipose tissues mainly due to enlarged adipocytes (Table 1, Figure 2 and 3). The weight of the liver was slightly higher in10 weeks old BFMI mice compared to B6 mice which could be due to increased fat accumulation in liver of BFMI mice (Table 1, Figure 3). Serum triglyceride concentrations, but not total cholesterol concentrations, were elevated in BFMI mice compared to B6.

**Table 1:** Body composition and serum parameters in 10 week old BFMI and B6 mice on SMD and BFMI mice on HFD

**Trait**	**B6**	**BFMI on SMD**	**BFMI on HFD**
	
Body weight (g)	23.4 (1.4)	39.2 (2.7)*	47.8 (2.3)+
			
Body composition traits			
Body lean mass (g)	19.97 (0.95)	29.29 (1.71)*	31.43 (1.38)+
Body fat mass (g)	0.97 (0.31)	8.84 (2.26)*	14.56 (2.26)+
Total white fat tissue (g)	1.11 (0.26)	7.70 (1.70)*	12.10 (0.95)+
Reproductive fat pad (g)	0.23 (0.05)	1.96 (0.43)*	2.26 (0.53)
Renal fat pad (g)	0.07 (0.03)	0.69 (0.13)*	1.01 (0.19)+
Subcutaneous fat pad (g)	0.50 (0.15)	2.57 (1.02)*	4.42 (1.42)+
Liver (g)	1.16 (0.15)	2.19 (0.22)*	3.33 (0.46)+
			
Serum parameter traits			
Triglycerides (mg/dl)	123 (63)	277 (95)*	245 (97)
Total cholesterol (mg/dl)	103 (20)	100 (11)	177 (24)+

SMD, standard maintenance diet; HFD, high-fat diet; values represent mean values with standard deviation in parenthesis of B6 on SMD (n = 15), BFMI on SMD (n = 11), and BFMI on HFD (n = 12). Serum parameters were obtained after a fasting period of two hours (BFMI on SMD (n = 8), BFMI on HFD (n = 8), and B6 (n = 12)). Asterisks indicate significant differences between BFMI860 and B6. Except for cholesterol all values were significantly different between B6 and BFMI mice on SMD. + indicates significant differences between standard maintenance and high-fat diet within line BFMI860 (P < 0.05).

Feeding of HFD in mice of the BFMI line caused an additional body weight and body fat mass gain (9.0 g and 6.4 g, respectively). The excess amount of white adipose tissue (WAT) was more pronounced in subcutaneous and renal adipose tissue than in the reproductive adipose tissues (Table 1). This is in accordance with the observation that subcutaneous white adipose tissue is known as a primary fat storage depot. However, adipocytes were only slightly increased in subcutaneous adipose and not further increased in reproductive and renal adipose tissue indicating that proliferation of adipocytes occurs to uptake the additional fat in HFD-fed BMFI mice (Figure 2). Liver weight was significantly increased in mice on HFD due to increased fat accumulation (Figure 3). Serum triglyceride concentrations were not further increased in BFMI mice on HFD, but serum total cholesterol concentrations were elevated (Table 1).

### Comparison of genes belonging to the PPAR pathway between BFMI and B6 mice

**Figure 4:**
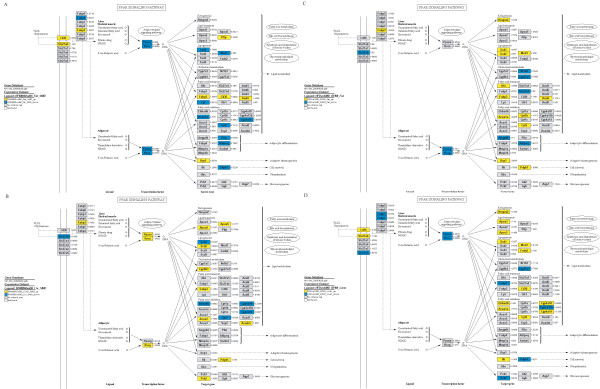
**Differentially expressed genes of the of *Ppar *pathway genes between BFMI and B6 on standard maintenance diet (SMD) (A) in reproductive adipose tissue (A) and liver (B) and between SMD- and high-fat diet (HFD)-fed BFMI mice in reproductive adipose tissue (C) and liver (D)**. The PPAR pathway is according to KEGG, visualisation was done with GenMapp.

Among the members of the PPAR signalling pathway (KEGG database), 20 and 19 genes were differentially expressed between the lines BFMI and B6 on SMD in adipose tissue and in liver, respectively (Table 2, Figure 4).

**Table 2:** Changes in expression of genes involved in the PPAR pathway.

	Gene symbol	BFMI860/B6 (SMD)				HFD/SMD (BFMI860)			
			
Entrez Gene-IDs		Fat		liver		fat		liver	
					
		Fold ch.	p-value	Fold ch.	p-value	Fold ch.	p-value	Fold ch.	p-value
PPAR isoforms and receptors									
19013	Ppara	0.87	< 0.01	2.67	< 0.01	0.95	ns	0.48	< 0.01
19015	Ppard	1.07	ns	0.98	ns	0.73	ns	0.88	ns
19016	Pparg	0.66	< 0.01	0.83	< 0.05	0.70	< 0.01	0.96	ns
20181	Rxra	0.69	< 0.01	1.09	< 0.05	0.95	ns	0.89	< 0.05
20182	Rxrb	0.98	ns	1.06	ns	1.00	ns	1.00	ns
20183	Rxrg	0.84	< 0.01	1.29	< 0.01	0.81	< 0.01	1.02	ns
Lipid transport									
11806	Apoa1	0.97	ns	0.96	ns	1.04	ns	1.11	< 0.01
11807	Apoa2	0.97	ns	1.21	< 0.05	1.16	ns	1.02	ns
66113	Apoa5	0.94	ns	1.32	< 0.05	1.00	ns	0.92	ns
11814	Apoc3	0.98	ns	0.94	ns	0.98	ns	1.15	< 0.01
18830	PLTP	2.32	< 0.01	0.75	ns	0.64	ns	1.26	ns
Fatty acid transport									
14081	Acsl1	0.97	ns	0.97	ns	0.82	ns	0.84	ns
74205	Acsl3	1.03	ns	1.01	ns	0.98	ns	0.94	ns
50790	Acsl4	1.14	< 0.05	0.98	ns	1.18	< 0.05	1.03	ns
433256	Acsl5	1.09	ns	1.02	ns	1.05	ns	1.12	ns
216739	Acsl6	0.97	ns	0.99	ns	1.01	ns	1.06	ns
12491	Cd36	1.61	< 0.01	1.06	ns	1.16	ns	1.42	< 0.05
13167	Dbi	0.86	ns	0.92	ns	1.39	< 0.01	1.08	ns
14080	Fabp1	1.09	ns	1.04	ns	0.97	ns	0.87	< 0.05
14079	Fabp2	1.05	ns	1.08	ns	0.95	ns	0.85	< 0.01
14077	Fabp3	1.36	< 0.05	1.15	< 0.05	1.83	< 0.01	0.90	ns
11770	Fabp4	1.00	ns	1.01	ns	1.26	ns	0.95	ns
16592	Fabp5	1.47	ns	0.88	ns	1.38	ns	0.99	ns
16204	Fabp6	0.94	< 0.01	1.02	ns	1.02	ns	1.04	ns
12140	Fabp7	1.07	ns	0.96	ns	0.97	ns	0.96	ns
16956	Lpl	0.85	< 0.01	1.02	ns	1.04	ns	0.95	ns
108078	Olr1	0.96	ns	1.01	ns	0.99	ns	0.95	ns
26457	Slc27a1	0.75	ns	0.97	ns	0.99	ns	1.03	ns
26458	Slc27a2	1.02	ns	0.97	ns	0.97	ns	1.15	ns
26569	Slc27a4	1.07	ns	1.01	ns	0.92	< 0.05	0.99	ns
26459	Slc27a5	0.99	ns	1.06	ns	1.00	ns	0.83	< 0.01
225579	Slc27a6	1.14	< 0.01	0.82	< 0.01	0.96	ns	1.11	< 0.05
Fatty acid oxidation									
113868	Acaa1a	0.78	< 0.05	0.97	ns	1.46	< 0.05	1.15	< 0.05
11363	Acadl	0.91	ns	1.1	ns	1.09	ns	1.18	ns
11364	Acadm	0.7	ns	1.89	< 0.01	0.93	ns	1.08	ns
11430	Acox1	1.07	ns	1.74	ns	1.06	ns	0.67	ns
93732	Acox2	1.05	ns	1.76	< 0.01	0.97	ns	0.80	ns
80911	Acox3	0.99	ns	1.01	ns	0.99	ns	0.99	ns
12894	Cpt1a	0.98	ns	1.01	ns	0.99	ns	0.94	ns
12895	Cpt1b	1.17	ns	0.99	ns	1.20	< 0.05	1.07	ns
78070	Cpt1c	0.96	ns	1.01	ns	0.97	ns	0.98	< 0.05
12896	Cpt2	0.71	< 0.01	0.63	< 0.01	1.61	< 0.05	1.85	< 0.01
13117	Cyp4A10	1.00	ns	0.24	< 0.01	0.98	ns	5.07	< 0.01
13118	Cyp4a12b	1.03	ns	0.80	ns	0.94	ns	0.59	< 0.05
13119	Cyp4a14	0.90	< 0.01	0.06	< 0.01	1.06	ns	31.29	< 0.01
74147	Ehhadh	0.98	ns	0.69	< 0.05	0.89	ns	2.17	< 0.01
20280	Scp2	0.85	ns	0.95	ns	0.87	ns	0.91	ns
Ketogenesis									
15360	Hmgcs2	0.92	ns	1.27	ns	1.72	< 0.01	1.14	< 0.05
Lipogenesis									
56473	Fads2	1.05	ns	0.69	ns	0.93	ns	3.16	< 0.01
677317	Mod1	0.58	< 0.01	0.75	ns	2.26	< 0.01	2.81	< 0.01
20249	Scd1	0.76	< 0.01	0.81	< 0.05	1.45	< 0.01	1.33	< 0.01
20250	Scd2	1.26	ns	1.46	< 0.01	2.03	< 0.01	1.16	ns
30049	Scd3	1.03	ns	0.98	ns	0.98	ns	1.02	ns
329065	Scd4	1.01	ns	1.06	ns	0.98	ns	1.00	ns
Cholesterol metabolism									
104086	Cyp27a1	1.04	ns	1.00	ns	0.78	< 0.01	0.70	< 0.01
13122	Cyp7a1	0.96	ns	2.31	ns	1.00	ns	0.49	ns
13124	Cyp8b1	1.00	ns	1.37	< 0.05	0.99	ns	1.04	ns
22259	Nr1h3	0.96	ns	0.92	ns	0.76	< 0.05	1.11	ns
Gluconeogenesis									
11832	Aqp7	0.83	ns	1.04	ns	1.12	ns	1.03	ns
14626	Gk2	1.02	ns	1.01	ns	1.05	ns	1.03	ns
14933	Gyk	1.01	ns	0.96	ns	1.00	ns	1.00	ns
18534	Pck1	0.65	ns	0.54	ns	0.87	ns	1.15	ns
74551	Pck2	0.99	ns	1.17	< 0.01	0.97	ns	0.86	< 0.05
Adipocyte differentiation									
11450	Adipoq	0.71	< 0.05	0.97	ns	0.49	< 0.01	1.01	ns
57875	Angptl4	1.41	ns	0.84	ns	0.71	< 0.01	1.04	ns
83995	Mmp1a	1.02	ns	0.99	ns	0.99	ns	1.00	ns
83996	Mmp1b	1.06	ns	0.97	ns	0.97	ns	1.04	ns
103968	Plin	0.70	ns	0.98	ns	0.68	ns	1.03	ns
20411	Sorbs1	0.95	ns	0.94	ns	1.04	ns	1.06	ns
Adaptive thermogenesis									
22227	Ucp1	2.65	< 0.01	1.03	ns	2.87	< 0.01	0.98	ns
Cell survival									
16202	Ilk	1.27	ns	0.83	ns	0.77	ns	1.14	< 0.05
18607	Pdpk1	0.62	< 0.01	1.38	< 0.01	1.61	< 0.05	0.83	< 0.05
Ubiquitination									
22190	Ubc	0.87	ns	1.25	ns	0.83	ns	0.76	ns

Changes in expression are expressed as fold changes in BFMI860 in comparison to B6 mice on SMD, and of HFD-fed BFMI860 mice compared to SMD-fed mice, respectively. SMD- standard maintenance diet, HFD- high-fat diet, ns-not significant

On SMD, the transcript amount of *Ppar-α*, *Ppar-γ *and their receptors *Rxra *and *Rxrg *in reproductive adipose tissue were lower in BFMI mice compared to B6. Correspondingly, down-stream activated genes of the fatty acid oxidation but also genes of the lipogenesis were down-regulated. Within the fatty acid oxidation pathway, members of the cytochrome family, the acetyl-CoA acyltransferase (*Acaa1*) and the carnitine palmitoyltransferase 2 (*Cpt2*) were decreased. Lipogenesis in BFMI mice was characterized by down-regulation of the malic enzyme (*Mod1*), which provides energy rich molecules for the fatty acid synthesis.

Transcript amounts of genes encoding phospholipid transfer protein (*Pltp*), *Cd36 *antigen, fatty acid binding protein (*Fabp3*) and Acyl-CoA-synthetase (*Acsl4*), which are responsible for lipid and fatty acid transport, were up-regulated in BFMI mice compared to B6. The uncoupling protein 1 (*Ucp1*) that enhances thermogenesis was also highly up-regulated in reproductive adipose tissue in BFMI mice.

Unlike in adipose tissue, the expression of *Ppar-α *and its receptors *Rxra *and *Rxrg *were increased in liver. As in adipose tissue, genes of the lipid or fatty acid transport (here *Apoa2*, *Apoa5 *and *Fabp3*) were more expressed in liver of BFMI mice than in B6 mice. Genes involved in fatty acid oxidation were differentially expressed between both mouse lines. Liver transcript amounts of Acyl-CoA oxidase (*Acox2*) and acyl-Coenzyme A dehydrogenase (*Acadm*) were higher, whereas 3-hydroxyacyl Coenzyme A dehydrogenase (*Ehhadh*), *Cpt2 *and members of the cytochrome P450 family were lower in BFMI than in B6.

### Diet effect on genes of the PPAR pathway in BFMI mice

To determine whether dietary fat regulates the expression of genes of the PPAR pathway in BFMI mice, mRNA expression profiles of reproductive adipose tissue and liver of mice on SMD and HFD were compared. In response to HFD, the mRNA expression of Ppar-γ and its receptor was down-regulated in reproductive adipose tissue, whereas the expression of *Ppar-α *and its receptor were reduced in liver. Transcriptional response in both tissues indicated that most genes involved in lipid metabolism were up-regulated (Table 2, Figure 4). An exception was the cholesterol metabolism, which was reduced. In particular, genes involved in lipogenesis (*Scd1*, *Mod1*) but also in peroxisomal and mitochondrial fatty acid oxidation (*Acaa1*, *Cpt2*) and in ketogenesis (*Hmgcs2*) were up-regulated in response to HFD.

In addition, the expression of genes of the fatty acid transport (*Acsl4*, *Dbi*, *Fabp3*) and of the cell survival (*Pdpk1*) were increased in reproductive adipose tissue. The Ucp1 expression was further elevated in line BFMI. Genes affecting adipocyte differentiation, like angiopoietin-like 4 (*Angptl4*), which is involved in the cellular response to starvation, and adiponectin were down-regulated in this tissue in response to HFD-feeding. In liver tissue, genes belonging to lipid transport (*Apoa1*, *Apoa3*) were further up-regulated.

## Discussion

The BFMI line is a model for studies of obesity and subsequent complications, which are close to obese humans. The purpose of the presented work was to evaluate the role of different *Ppar *genes and their responsive genes in the fat accumulation in the BFMI mice as PPARs are important regulators of lipid metabolism and energy homeostasis. Increased fat accumulation accompanied with increased adipocyte size and elevated serum triglyceride levels, observed in BFMI compared to B6 mice in this study, makes a change of *Ppar *gene expression and their responsive genes very likely. As PPAR-α agonists lead to reduced adiposity [4,5] and PPAR-γ agonists stimulate lipid storage and body weight gain [7,8] we would have expected a down-regulation of *Ppar-α *in liver and an up-regulation of *Ppar-γ *in adipose tissue in BFMI mice compared to B6. In contrast to our expectation, transcript amounts in white adipose tissue and in liver revealed a down-regulation of *Ppar-α *and *Ppar-γ *and their receptors in adipose tissue and an up-regulation of *Ppar-α *and its receptor in liver in the high-fatness selected BFMI line compared to B6. In response to HFD, BFMI mice showed a further reduction of *Ppar-γ *and *Rxrg *mRNA levels in adipose tissue but a reduced mRNA level of *Ppar-α *and its receptor in liver.

The observation that *Ppar-α *mRNA levels are increased in liver of BFMI mice compared to B6 corresponds well with findings in *ob/ob *and *db/db *mice in comparison with lean mice [12]. But this is in contrast with findings in *Ppar-α *deficient mice which had higher fat mass, liver weight and triglyceride concentrations [13,14]. In obese mice, PPAR-α might be activated to counteract the obese state. In BFMI mice, PPAR responsive genes involved in lipid transport are up-regulated, but despite the up-regulation of PPAR-α expression genes involved in fatty acid oxidation are down-regulated, which leads to fat accumulation in the liver. The accumulation of fat in the liver suggests preferred usage of glucose for oxidation, which is consistent with higher respiratory quotients in BFMI mice [10].

BFMI mice responded to HFD by a reduction of PPAR-α expression in liver. This is in line with increased adiposity in *Ppar-α *null mice after HFD-feeding [4]. Our finding is in contrast with the increased PPAR-α expression in diet-induced obese B6 mice, in which it goes along with elevated fatty acid oxidation [14]. In BFMI mice, genes of lipid transport, lipogenesis, fatty acid oxidation and ketogenesis were up-regulated despite the reduced *Ppar *expression. This finding suggests hat the PPAR-α signalling in BFMI mice might be disturbed or that other factors have a stronger influence on lipid metabolism. Despite elevated fatty acid utilization in the liver the high lipid storage resulted in a fatty liver in HFD-fed BFMI mice.

PPAR-γ activation is essential for the formation of adipocytes and, as a consequence, *Ppar-γ *knockout mice fail to develop adipose tissue [7,8]. In *db/db *and *ob/ob *mice PPAR-γ agonism or activation led to the reduction of serum concentrations of triglycerides [5,15]. Surprisingly, in BFMI mice *Ppar-γ *is down-regulated despite high body fat mass and increased adipocyte size in comparison to B6. In the white adipose tissue, the decreased *Ppar-γ *expression in BFMI mice goes along with decreased fatty acid oxidation. This coincides with the observation in Zucker rats, in which PPAR-γ agonism led to stimulation of genes involved in fatty acid oxidation [16]. In BFMI mice, reduction of fatty acid oxidation might be also due to the interaction with *Ppar-α*, which is also lowered in adipose tissue. Reduced fatty acid oxidation and elevated fatty acid transport in BFMI mice compared to B6 lead to an elevated fat deposition and increased white adipose tissues with hypertrophic adipocytes in BFMI mice. As the proportion of the adipose tissue weights between BFMI and B6 mice was larger than the proportion of the adipocyte size between the lines, we suggest that proliferation of adipocytes still occurs in BFMI mice.

However, there were also fat storage counteracting effects in BFMI mice. The expression of *Ucp1*, which facilitates thermogenesis, was highly increased in line BFMI in comparison to B6. UCPs could dispose excess energy by uncoupling mitochondrial respiration from oxidative phosphorylation under fatty acid consumption [17]. In response to HFD, *Ucp1 *as well as genes of the fatty acid oxidation (*Acaa1a*, *Cpt1b*, *Cpt2*) were further elevated in adipose tissue of BFMI mice. Moreover, in adipose tissue and liver of BFMI mice on HFD, fatty acids seem to be converted to ketones as energy sources. The high amount of *Ucp1 *transcripts and the change of fatty acid utilisation indicate a counteraction to the excessive energy supply and might explain the unchanged triglyceride levels in BFMI mice on SMD and HFD. An up-regulation of *Ucp1 *in response to HFD was also seen in DBA/2J mice [18]. The increase in fat mass in HFD-fed mice mirrors further lipid storage as a result of fatty acid uptake and lipogenesis which were up-regulated. As the adipocyte size was not further increased, but tissue mass nearly doubled in HFD-fed BFMI mice compared to SMD, cell proliferation still takes place, thus developing additional adipocytes. This is indicated by an up-regulation of *Pdpk1*, which is involved in cell survival. Surprisingly, new adipocytes are developed in BFMI mice despite the decreased *Ppar-γ *expression in response to HFD. This is in contrast with findings in heterozygous *Ppar-γ *mutant mice that had smaller fat pads [19]. In BFMI mice, down-regulation of *Ppar-γ *expression seems to counteract fat accumulation rather than to promote fat storage.

PPAR-δ is abundantly expressed throughout the body, but little is known about its role in lipid metabolism [2]. Although it seems to play a role in obesity [20], *Ppar*-δ expressions of reproductive adipose tissue and liver were similar in BFMI and B6 mice. Furthermore, they were not changed by the diet. The same was found in liver of *ob/ob *and *db/db *mice in comparison to B6 [12].

In summary, elevated fatty acid and lipid transport into adipose tissue accompanied by lower fatty acid oxidation contribute to hypertrophy of adipocytes and excess of body fat mass in the high-fatness selected BFMI line. Our results suggest that the regulation of *Ppar *genes in BFMI mice is a strategy of the organism to counteract the rise of obesity rather than promoting the formation of the obese phenotype. However, the signalling from *Ppar *genes to the fatty acid oxidation seems to be disturbed. In response to HFD, likewise mechanisms seem to be activated that hamper fat gain. In HFD-fed BFMI mice, the fatty acid oxidation, ketogenesis and thermogenesis are up-regulated indicating increased utilization of fatty acids as energy source. Despite increased fatty acid oxidations, elevated lipogenesis led to additional fat stored in the adipose tissue and fatty liver in HFD-fed BFMI mice.

Considering the many differences in PPAR gene expression in comparison to B6 mice and in the response to HFD, BFMI mice provide a new model for the study of PPAR actions under controlled genetic and environmental conditions.

## Methods

### Animals and diets

In this study, we used males of the BFMI860 line which was generated from an outbred population. Founders of the BFMI line were originally purchased from pet shops and were consecutively selected for low protein content, low body mass and high fat content, and for high fatness for 58 generations before inbreeding [9]. C57BL/6NCrl (B6) mice (Charles River Laboratories, Sulzfeld, Germany) were used as a control because this mouse line was a parental line for a cross-bred experiment to map genetic loci affecting body fat content in BFMI mice [11]. Mice were kept at room temperature (22°C - 24°C) with a light dark cycle of 12 hours. After weaning at the age of three weeks, 11 mice of the BFMI line and 15 mice of the B6 line were randomly chosen and fed a standard maintenance diet (SMD) containing 12.8 MJ/kg metabolizable energy with 9% of its energy from fat, 33% from protein content and 58% from carbohydrates (V1534-000 ssniff R/M-H, ssniff Spezialdiäten GmbH, Soest, Germany). Twelve additional males of the BFMI line were set on a high-fat diet (HFD) containing 19.1 MJ/kg metabolizable energy with 45% of its energy from fat, 24% from protein content and 31% from carbohydrates (S8074-E010 ssniff EF R/M, ssniff Spezialdiäten GmbH, Soest, Germany). The ingredients of both diets are given in Table 3. The standard diet derived its fat from soy oil, whereas the high-fat diet derived its fat from coconut oil and suet. Animals had *ad libitum *access to water and diets. All animal treatments were in accordance with the German Animal Welfare Legislation (approval no. G0152/04).

**Table 3:** Ingredients of standard maintenance diet (SMD) and high-fat diet (HFD)

Ingredient	SMD	HFD
Crude nutrients	proportion [%]	
Crude proteins	19.0	20.7
Crude fat	3.3	25.0
Crude fiber	4.9	5.0
Crude ashes	6.7	5.9
Dry substance	87.7	96.3
N-free extracts	54.1	39.7
Starch	36.5	20.0
Sugar	4.7	17.5

Metabolizable energy	MJ/kg	
	12.8	19.1
	% of energy	
Fat	9.0	45.0
Carbohydrate	58.0	31.0
Protein	33.0	24.0

Both diets contained also vitamins, trace elements, and minerals.

### Phenotypes

For the phenotypic characterization, animals were weighed weekly on the basis of their day of birth from the age of 3 weeks on. Body fat mass and body lean mass were also determined weekly in non-anesthetized animals by quantitative magnetic resonance (QMR) analysis using the EchoMRI whole body composition analyzer (Echo Medical Systems, Houston, Texas, USA) [21]. At 10 weeks, mice were fasted for two hours, anesthetized with isofluran and immediately killed by decapitation. After bleeding, different white adipose tissues (reproductive, renal, subcutaneous) and liver were dissected and weighed. The remaining white adipose tissues were collected and weighted. The sum of all visible white adipose tissues was termed 'total white fat tissue'.

### Histology

For the histological examinations, the reproductive adipose tissue (around the testicles), the retroperitoneal (as part of the renal adipose tissue), the inguinal adipose tissue (as part of the subcutaneous adipose tissue) and the liver of three animals of each line and diet group were immediately fixed upon dissection in 4% buffered formaldehyde solution (pH 7.4) for 24 hours. Adipose tissue samples were embedded in paraffin following standard laboratory procedures. Sections (3 μm) of dewaxed tissues were stained with hematoxylin and eosin. Adipocyte cell diameter was measured from 30 cells per tissue per mouse. Cells with a round shape were chosen for determination of the cell size. Cryosections (10 μm) of liver tissue were incubated with 0.18% oil-red O for 10 min, washed with 60% isopropanol, and counterstained with hematoxylin. Sections were photographed at 100x magnification with a digital camera.

### Serum parameters

Blood was collected at slaughtering and serum was recovered by centrifugation for 15 min at 600 g and stored at -20°C until analysis. Serum triglycerides and total cholesterol were determined using the Fluitest TG (Triglyceride GPO-PAP) and Fluitest Chol (Cholesterin CHOD-PAP) kits (both Biocon Medizintechnik, Mönchberg, Germany).

### RNA isolation and microarray analysis

For gene expression analyses, the reproductive adipose tissue and liver of six mice per feeding group at ten weeks were immediately frozen in liquid nitrogen. We used the reproductive adipose tissue for gene expression analysis as visceral white adipose tissue is known to be more metabolically active than subcutaneous [18,22]. Total RNA was prepared using the NucleoSpin RNA II Kit (Macherey-Nagel, Düren, Germany) following the manufacturer's protocol. Quality of RNA was verified on an agarose gel with ethidium bromide staining. 400 ng of RNA was reverse transcribed to synthesize first and second strand complementary DNA (cDNA), purified with spin columns and then in vitro transcribed to synthesize biotin-labelled complementary RNA (cRNA) (Ambion Illumina RNA Amplification Kit, Cambridgeshire, UK). 1.5 μg biotin-labeled cRNA was hybridized onto Mouse-6 v1 Expression BeadChips (Illumina Inc, San Diego, CA, USA), representing >46,000 probe sequences per array, for 16 hours at 55°C. BeadChips were then scanned using Illumina's BeadStation 500X gene expression system (Illumina Inc., San Diego, CA). Fluorescence images of the Illumina BeadArrays were translated into relative expression levels using the Bioconductor [23] package beadarray [24] with standard parameter settings. The intensity values of the arrays were log2-transformed and quantile-normalized for each tissue separately.

### Analyses of expression data

ANOVA was performed to evaluate differences between (i) the BFMI and B6 mice on SMD and (ii) between BFMI mice on SMD and HFD using the SAS 9.1 statistical software package (SAS Institute Inc., Cary, NC, USA). For body composition and serum parameters, the Wilcoxon rank sum test was used and for the comparison of the adipocyte diameters, t-tests for two group comparisons were used. P-values smaller than 0.05 were considered statistically significant. For the comparison of time courses for weight and fat gain, longitudinal data were subjected to ANOVA with repeated measurement.

To measure the significance of differential expression two separate t-tests were performed for each oligo in each tissue: BFMI on SMD vs. B6 on SMD and BFMI on HFD vs. BFMI on SMD. To address multiple testing, local false discovery rates (local FDR) were calculated. For this purpose a customized algorithm following Aubert et al. [25] was implemented in R. Differentially expressed genes which had both p-values smaller than 0.05 and local FDRs smaller than 0.5 were considered statistically significant. Where necessary, medians of fold-changes and medians of significance values (p-value and local FDR) were used to define unique values for each gene if multiple oligos were present on the chip that measured the expression of the same gene. Genes showing significant differences between groups were assigned to the PPAR pathway based on the Kyoto Encyclopedia of Genes and Genomes (KEGG) database [26]. Pathways were visualized with GenMAPP [27].

## Competing interests

The authors declare that they have no competing interests.

## Authors' contribution

AW planned the mouse experiments and drafted the manuscript. HFG performed the analyses of the microarray data. AOS participated in the analyses of the microarray data and the statistical analyses. SM prepared the histological staining. ADG participated in the histological studies. RR performed the chip analysis. GAB designed the BFMI model, coordinated the study and contributed to the manuscript. All authors read and approved the final manuscript.
